# Characterization and Photocatalytic and Antibacterial Properties of Ag- and TiO_x_-Based (x = 2, 3) Composite Nanomaterials under UV Irradiation

**DOI:** 10.3390/ma17102178

**Published:** 2024-05-07

**Authors:** Nicola Morante, Veronica Folliero, Federica Dell’Annunziata, Nicoletta Capuano, Antonietta Mancuso, Katia Monzillo, Massimiliano Galdiero, Diana Sannino, Gianluigi Franci

**Affiliations:** 1 Department of Industrial Engineering, University of Salerno, Via Giovanni Paolo II, 132, 84084 Fisciano, SA, Italy; nmorante@unisa.it (N.M.); anmancuso@unisa.it (A.M.); kmonzillo@unisa.it (K.M.); 2 Department of Medicine, Surgery and Dentistry, University of Salerno, 84084 Baronissi, SA, Italy; vfolliero@unisa.it (V.F.); federica.dellannunziata@unicampania.it (F.D.); niccapuano@unisa.it (N.C.); 3 Department of Experimental Medicine, Section of Microbiology and Clinical Microbiology, University of Campania “Luigi Vanvitelli”, 80138 Naples, NA, Italy; massimiliano.galdiero@unicampania.it

**Keywords:** nanostructured materials, antibacterial activity, titanium dioxide, silver nanoparticles

## Abstract

Metal and metal oxide nanostructured materials have been chemically and physically characterized and tested concerning methylene blue (MB) photoremoval and UV antibacterial activity against *Escherichia coli* and *Staphylococcus aureus*. In detail, silver nanoparticles and commercial BaTiO_3_ nanoparticles were modified to obtain nanocomposites through sonicated sol–gel TiO_2_ synthesis and the photodeposition of Ag nanoparticles, respectively. The characterization results of pristine nanomaterials and synthetized photocatalysts revealed significant differences in specific surface area (SSA), the presence of impurities in commercial Ag nanoparticles, an anatase phase with brookite traces for TiO_2_-based nanomaterials, and a mixed cubic–tetragonal phase for BaTiO_3_. Silver nanoparticles exhibited superior antibacterial activity at different dosages; however, they were inactive in the photoremoval of the dye. The silver–TiO_x_ nanocomposite demonstrated an activity in the UV photodegradation of MB and UV inhibition of bacterial growth. Specifically, TiO_2_/AgNP (30–50 nm) reduced growth by 487.5 and 1.1 × 10^3^ times for *Escherichia coli* and *Staphylococcus aureus*, respectively, at a dose of 500 μg/mL under UV irradiation.

## 1. Introduction

Healthcare-associated infections (HAIs) represent a significant challenge for hospitalized patients, resulting in both clinical and economic consequences [1]. The main individuals susceptible to these infections are patients, but healthcare workers, volunteers, trainees, and students are also at risk [2]. A recent in-depth investigation conducted by the European Center for Disease Prevention and Control (ECDC) reveals alarming statistics in Europe [3]. In detail, 3.2 million patients are affected by at least one HAI, with 37,000 direct deaths. Italy, specifically, reports 450–700,000 new cases per year. According to the latest data, there are approximately 426,277 cases of HAIs caused by antimicrobial-resistant microorganisms annually in the European Union [4]. The European Center for Disease Prevention and Control (ECDC) outlines the top ten frequently isolated microorganisms associated with HAIs, such as *Staphylococcus aureus* (*S. aureus*), *Enterococcus* spp., *Escherichia coli* (*E. coli*), *Klebsiella* spp., *Enterobacter* spp., *Pseudomonas aeruginosa*, and *Acinetobacter baumannii* [5]. In cases of HAI, 20–40% can be attributed to the patient’s internal flora, but the transmission of microorganisms goes beyond endogenous factors. Environmental elements, such as air, water, and surfaces (patient rooms, medical equipment, etc.), are fundamental in this process [6]. Recent scientific revelations highlight the critical role played by inanimate surfaces in the spread of multidrug-resistant nosocomial pathogens [7,8]. To ensure a safe environment, it is essential to constantly clean and disinfect surfaces, paying particular attention to those commonly touched by both patients and healthcare personnel. The research has highlighted cases of non-compliance with disinfection and sterilization protocols established in healthcare facilities [9,10]. Therefore, there is an urgent need to intensify research in the field of nanostructured metallic materials to reduce microbial contamination on various surfaces. Metal and metal oxide nanostructured materials (Me-NMs and MeO-NMs) have garnered significant interest owing to their extensive antibacterial activity with a broad spectrum [11]. Among Me-NMs, silver nanoparticles (AgNPs) are known for their significant broad-spectrum antibacterial property [12]. AgNPs host from 20 to 15,000 silver atoms, with a diameter typically less than 100 nm [13]. AgNP synthesis can be achieved through physical, chemical, and biological methods. Physical methods involve processes, such as evaporation–condensation, laser ablation, and ball milling, which offer control over the size and shape of nanoparticles but require high energy and specialized equipment. Chemical methods include silver salt reduction, chemical precipitation, and micro-emulsification, providing high yield and controlled properties but involving the use of hazardous chemicals and generating toxic byproducts. Biological methods employ plant extracts, microorganisms, or enzymes to facilitate the reduction of silver ions to nanoparticles, presenting a number of advantages as well as some limitations. Although disadvantages include prolonged synthesis durations and batch-to-batch variability, resulting from limited control over nanoparticle properties, numerous advantages abound. These include environmental sustainability, cost reduction, the production of biocompatible nanoparticles, and the avoidance of toxic substances [14]. AgNPs exhibit distinctive photocatalytic and antibacterial properties. The photocatalytic activity of silver nanoparticles is primarily attributed to their ability to generate reactive oxygen species (ROSs) under light irradiation. This process involves the excitation of surface plasmon resonance (SPR) in the nanoparticles, leading to the generation of electron–hole pairs. These electron–hole pairs can react with adsorbed oxygen and water molecules to produce ROSs, such as hydroxyl radicals, superoxide radicals, and singlet oxygen. These ROSs possess high oxidative potential and can effectively degrade organic pollutants and inhibit the growth of microorganisms [15]. AgNPs are recognized for their robust antibacterial activity. Their high antibacterial activity derives from multiple mechanisms of action, which provide a broad spectrum of action and a reduced propensity to develop resistance [16]. AgNPs induce the formation of pores in the cell wall, altering its structure and permeability. Furthermore, in the cytoplasmic district, they denature ribosomes, interfere with DNA replication, and interrupt the production of ATP. MeO-NMs emerge as coveted disinfection agents owing to their remarkable durability, enhanced stability, and minimal toxicity to eukaryotic cells, unlike their metallic counterparts [17,18,19]. Among MeO-NMs, TiO_x_-based nanomaterials (the main TiO_2_ and interesting BaTiO_3_) exhibit distinctive chemical and physical properties that enable them to exert increased bacterial toxicity, primarily attributed to their remarkable photocatalytic properties. These characteristics of MeO-NMs are relevant for achieving disinfection [20]. Photocatalysis with MeO-NMs belong to heterogeneous photocatalysis, wherein the activation of the catalyst occurs through the transfer of the appropriate activation energy via radiation [21]. When photons with energy equal to or greater than that of the bandgap (BG) of the nanoparticles (NPs) are absorbed, electrons are promoted from the valence band (VB) to the conduction band (CB), leaving behind a positive hole in the VB. The resulting pair of separate charges, formed one per photon and lasting for a few nanoseconds, migrates on the mass and the surface of the photocatalyst and can participate in redox reactions with species adsorbed on the surface. If they meet during migration, they recombine in the form of heat, but this recombination must be considered as being a secondary reaction because the energy of the photons is lost in the reaction. This mechanism explains the formation of reactive oxygen species (ROSs), which are formed by e^−^-h^+^ pairs and through the interactions of water or oxygen, such as OH·, O_2_^−^·, OOH·, and H_2_O_2_, as the main ones [22]. ROSs play a fundamental role in causing DNA degradation, oxidative damage to polyunsaturated fatty acids, and the deterioration of amino acids through oxidative processes [23]. To mitigate the effects of ROS-induced oxidative stress, bacteria employ various defense mechanisms [24]. Among these, the most explored are enzymes that eliminate ROSs, including superoxide dismutase, catalase, and peroxidase [24]. The induction of oxidative stress is not the only mechanism of Me-NMs. These nanomaterials also alter the structure of cell walls. The negatively charged polysaccharides of the bacterial membrane interact with the metal cations released by the Me-NMs, resulting in their accumulation on the cell surface. This, in turn, damages the structure and permeability of the bacterial wall [25]. In addition, Me-NMs alter the balance of intracellular metal ions, which are crucial for microbial survival. These ions play a crucial role in metabolic functions and aiding coenzymes and cofactors. Me-NMs slowly release metal ions through adsorption, dissolution, and hydrolysis. When there is an excess of bacteria, metabolic functions are impaired. Metal ions bind to DNA, altering its structure [26]. Evidence has documented the ability of these nanomaterials to destroy proteins and enzymes. Metal ions catalyze the oxidation of amino acid side chains, resulting in protein-bound carbonyls. This carboxylation causes the loss of their catalytic activity. Furthermore, MeO-NMs also interfere with signal transduction [27]. Phosphotyrosine, mainly involved in bacterial signal transduction, undergoes dephosphorylation, blocking signal transduction and, consequently, bacterial growth [28]. The most common and well-known Me-NPs are silver nanoparticles (AgNPs). They are now produced and sold. As the process of disinfection is a large-scale issue, these extensively produced nanoparticles can help to ensure disinfection if properly applied. However, being a high-cost metal, it is necessary to reduce the amount used and avoid nanoparticle aggregation during applications. Two strategies can be adopted: the formation of a suitable composite or dispersion on active supports.

Therefore, this study aims to exploit the antibacterial activity of commercial AgNPs and their application in combination with TiO_x_-based nanomaterials. This can reduce the amount of AgNPs used, increasing the stability and safety of the designed nanomaterial. Furthermore, the combination of TiO_2_ and AgNPs offers the additional advantage of having a co-catalyst (Ag) that can limit the recombination of photogenerated electron–hole pairs, which is the main disadvantage of the TiO_2_ photoactivity alone. A final aspect is linked to the presence of silver in the nanocomposites, which can act as antibacterial storage. To overcome the known issues of nanoparticle aggregation, photodeposited silver nanoparticles on BaTiO_3_ will be studied. To the best of the authors’ knowledge, photodeposited Ag on BaTiO_3_ NPs is still not reported elsewhere in the literature. 

Another important application of silver and titania nanoparticles is the treatment of water containing dyes [29,30,31]. Indeed, as reported in the literature, both Ag and TiO_2_ are active under solar light for the degradation of methylene blue [32,33].

The TiO_x_-based nanomaterials will be tested for their photocatalytic activity in methylene blue (MB) removal, while the antibacterial activity of the selected nanomaterials will be evaluated against *S. aureus* and *E. coli*, the main nosocomial agents for which it is essential to limit their spread.

## 2. Materials and Methods

### 2.1. Nanomaterials

The studied nanomaterials were partly acquired and partly synthesized by controlled production, as detailed below.

Commercial AgNPs (purity: 99.9%) of two declared size ranges, 30–50 nm and 50–80 nm, along with BaTiO_3_ powders (purity: >99%, average particle size: 100 nm) were obtained from Nanoshel UK LTD. (Cheshire, UK). Some details, including SEM images, particle size distribution, appearance, and density, can be found at the following website: https://nanoshel.com/product, accessed on 10 April 2024.

### 2.2. Chemicals 

Sigma Aldrich (St. Louis, MO, USA) provided the following products: isopropanol ((CH_3_)_2_CHOH, 99% (*w*/*w*)), methylene blue (C_16_H_18_ClN_3_S, 99.95% (*w*/*w*)), titanium tetraisopropoxide (C_12_H_28_O_4_Ti, greater than 97% (*w*/*w*)), and silver nitrate (AgNO_3_ ≥ 99% (*w*/*w*)). Water from MilliQ (Darmstadt, Germany) was utilized. The reagents were used exactly as supplied. 

### 2.3. Synthesis of TiO_2_ and TiO_2_–Silver Nanoparticle Composite

The visible-light-active TiO_2_-based photocatalytic nanoparticles were synthesized using a modified sol–gel procedure, introducing sonication to the methodology outlined in our earlier work [34]. We employed a modified wet chemical approach to synthesize both TiO_2_ and an innovative TiO_2_/Ag nanocomposite. In detail, the nanocomposite was obtained by dispersing 1 g of AgNPs in 50 mL of MilliQ water, followed by sonication of the suspension for 10 min. Subsequently, 12.5 mL of titanium tetraisopropoxide (TTIP) was continuously added to the suspension while being held in a sonic bath to prevent the sedimentation of AgNPs. This process resulted in a nominal weight percentage of 40 wt.% AgNPs. A similar procedure, but in the absence of AgNPs, was used for the sonicated-TiO_2_ preparation. The resulting powders, after washing and centrifugation, were calcined at 450 °C for 30 min.

### 2.4. Ang Photodeposition on BaTiO_3_NPs

To achieve a silver loading of 0.25 wt.% over BaTiO_3_ particles, 6.25 mL of isopropanol, as a sacrificial agent, and the remaining amount of the MilliQ water were added to a 250 mL flask [35]. The resulting suspension was then transferred to a beaker containing the required amount of the photocatalyst and subjected to sonication for 10 min at 20 °C in an ultrasonic bath at 99% power. Subsequently, the suspension was transferred to a crystallizer and agitated for ten minutes in a nitrogen-filled environment. After that, the solution was continuously stirred and exposed to a N_2_ flux for two hours using two 8 W UV lamps, each with a light intensity of 30 mW cm^−2^. After centrifuging the suspension, the resulting solid was dried for eight hours at 90 °C. Noble-metal-reduced nanoparticles were used to enhance the separation of photogenerated charges, as they can pull electrons.

### 2.5. Raman Spectra Acquisition

The instrument for the Raman spectra acquisition was an integrated confocal micro-Raman system (Invia Micro-Raman, Renishaw, Wotton-under-Edge, UK) equipped with three internal lasers as excitation wavelength sources: Nd:YAG (514 nm), HeNe (632.8 nm), and a diode (785 nm). The spectrometer has Notch filters for the rejection of the Rayleigh excitation line. Measurements were performed using radiation at 514 nm, with a real output power of 25 mW; the spectra were acquired on focalized powder samples at a 20× magnitude.

### 2.6. Specific Surface Area Measurements

The specific surface area (SSA) was analyzed from dynamic N_2_ adsorption at a low temperature (−196 °C) with a Costech Sorptometer 1042 instrument (CostechInternational s.r.l., Pioltello (MI), Italy) and powder samples. Prior to the measurements, the powder samples were pretreated at 150 °C for 30 min in a He flow.

### 2.7. UV-Vis DRS and Elaboration

Ultraviolet–visible diffuse reflectance spectroscopy (UV–vis DRS) was employed to determine the light absorption properties of the synthesized photocatalysts. Reflectance spectra were obtained through a Perkin–Elmer spectrophotometer Lambda 35 (PerkinElmer, Waltham, MA, USA), equipped with an 88-sample positioning holder (Labsphere Inc., North Sutton, NH, USA). The reflectance data were reported in terms of Kubelka–Munk values (F(R∞)) as a function of the wavelength. The indirect bandgap of the samples was determined by plotting (F(R∞) hν)^0.5^ vs. hν (eV), with the exception of the AgNP samples, AgNP 30–50 nm and AgNP 50–80 nm, for which the bandgap calculation was carried out using the direct method.

### 2.8. Thermal Analysis

Thermal gravimetric analysis, derivative thermogravimetry, and differential scanning calorimetry (TGA-DTG-DSC) are the thermal techniques used to measure the weight change and heat flow in a material as functions of the temperature and time in a controlled environment. Thermal analysis was conducted using a TG analyzer—Q600, TA Instrument (New Castle, DE, USA), on powder samples under an airflow of 100 STP mL min^−1^ and with a heating rate of 10 °C min^−1^ in the temperature range 25–900 °C.

### 2.9. XRD Analysis and Crystallite Size Evaluation

An automatic Bruker D8 Advance diffractometer (VANTEC-1 detector, Bruker, Billerica, MA, USA) with reflection geometry and nickel-filtered Cu-Kα radiation were used for the acquisition of X-ray diffraction (XRD) spectra. The crystallite size of the samples was calculated using the Debye–Scherrer equation as follows [36]:

(1) $$t=\frac{K\;\lambda }{\beta \cos\theta }$$

where K is the Scherrer constant of 0.89, λ indicates the wavelength of the X-ray source, β is the full width at half maxima (FWHM), and θ corresponds the Bragg diffraction angle.

### 2.10. Experimental Apparatus for the Photocatalytic Tests

The photocatalytic activity tests for the mineralization of methylene blue (MB) were conducted in a batch photoreactor comprising a cylindrical quartz container with the following dimensions: (i) internal diameter = 10 cm and (ii) height = 6 cm. The solution to be treated was prepared by dissolving an appropriate mass of the dye in 100 mL of distilled water. In particular, the tests were carried out using an initial concentration of MB equal to 7 ppm and a photocatalyst dosage of 3 g L^−1^ and operating at the spontaneous pH of the solution (5.5). To ensure the continuous mixing of the suspension, the reactor was placed on a magnetic plate, setting the stirring at 300 rpm. Two UV-A lamps with a peak emission at 365 nm were used as light sources (nominal power: 8 W; light intensity: 35 mW cm^−2^) to activate the photocatalytic particles. To prevent the heating of the reaction system because of the lamps, a fan was placed next to the photoreactor for cooling. Additionally, to prevent the dispersion of the light, the irradiation sources (positioned above the reactor at a distance of 15 cm) were covered with aluminum foil. The schematic picture of the experimental apparatus is reported in Figure S1. Before the light phase, during which the lamps were turned on to activate the photocatalyst, a dark phase (2 h) was conducted to ensure that the adsorption/desorption equilibrium of the dye molecules was reached on the surface of the dye. Throughout the tests, samples of the treated suspension (4 mL) were collected at specific time intervals during both the dark and light phases to assess the adsorption and degradation/mineralization of the MB. In particular, to evaluate the concentration of the azo dye, a UV-vis spectrophotometer (Thermo Scientific Evolution 201, Waltham, MA, USA) was used to analyze the absorbance’s value at 663 nm for the collected samples [37]. Furthermore, to calculate the mineralization of the pollutant and its intermediates formed during its photocatalytic oxidation, the TOC of the samples was determined by measuring the parts per million of CO_2_ produced through combustion using a high-temperature catalytic combustion method with a Pt/Al_2_O_3_ catalyst in a cylindrical fixed-bed reactor operating at 680 °C [35].

The azo dye discoloration was studied by a kinetic analysis using the Langmuir–Hinshelwood model [37,38], for which the discoloration rate (*r*) is expressed as follows:

(2) $$r=\frac{k_{r}\;K_{ad}\;c}{1+K_{ad}\;c}$$

where *k_r_*, *K_ad_*, and *c* are the kinetic constant for MB discoloration, adsorption equilibrium constant, and concentration of the dye, respectively. 

Equation (2) can be condensed to the following first-order kinetic expression with an apparent discoloration kinetic constant (*k_disc_*), assuming the adsorption is weak and the pollutant’s concentration is low:

(3) $$r=k_{r}\;K_{ad}\;c=k_{disc}\;c$$

Through the mass balance of the reaction system, it is possible to obtain the following expression:

(4) $$\frac{dc}{dt}=-k_{disc}\;c$$

Integrating Equation (4) between the initial time (*t* = 0) and the generic irradiation time (*t*), it is possible to calculate the apparent kinetic constant as follows:

(5) $$-\int_{c_{0}}^{c}\frac{1}{c}\;dc=k_{disc}\;\int_{0}^{t}dt$$

(6) $$F\left(t\right)=-\ln\left(\frac{c}{c_{0}}\right)=k_{disc}\;t$$

Similarly, assuming that mineralization follows first order kinetics, the following relationship is obtained:

(7) $$G\left(t\right)=-\ln\left(\frac{TOC}{{TOC}_{0}}\right)=k_{min}\;t$$

where *k_min_* is the apparent mineralization kinetic constant.

The slopes of the lines formed by plotting −ln(*c*/*c*_0_) vs. time and −ln(*TOC*/*TOC*_0_) vs. time provide *k_disc_* and *k_min_* values, respectively. Moreover, the following relationships were used to calculate the TOC removal (mineralization) and MB discoloration efficiency at a given irradiation time:

(8) $$TOC\;removal\;efficiency\;\left(t\right)=\left(1-\frac{TOC\left(t\right)}{TOC_{0}}\right)\;100$$

(9) $$MB\;discoloration\;efficiency\;\left(t\right)=\left(1-\frac{c\left(t\right)}{c_{0}}\right)\;100$$

where *TOC*(*t*) is the total organic carbon at the generic irradiation time (mg L^−1^), *TOC*_0_ is the initial total organic carbon (mg L^−1^), *c*(*t*) is the MB concentration at the generic irradiation time (mg L^−1^), and *c*_0_ is the initial MB concentration (mg L^−1^).

### 2.11. Bacterial Growth Conditions

The antibacterial properties of various nanomaterials were assessed using reference strains of *S. aureus* (ATCC 6538) and *E. coli* (ATCC 11229), acquired from the American Type Culture Collection (Manassas, VA, USA). They were grown in Mueller–Hinton (MH) medium (Oxoid, Basingstoke, Hampshire, MA, USA) at 37 °C under aerobic conditions. The bacterial inoculum required for antibacterial assays was obtained by inoculating fresh colonies of each strain, grown on MH agar, in MH medium and incubated at 37 °C overnight. Afterward, the bacterial suspension was diluted in fresh medium and incubated at 37 °C until reaching the exponential phase. Serial dilutions were performed to achieve the bacterial load suitable for antibacterial assays (1 × 10^6^ CFU mL^−1^).

### 2.12. Antibacterial Activity Evaluation

The test was conducted in sterile 48-well plates, in which the compounds were diluted to a final volume of 250 μL for concentrations of 500, 100, 50, and 10 μg/mL. Ampicillin and vancomycin were used as negative controls (CTR−) for *E. coli* and *S. aureus*, respectively. Otherwise, the bacterial suspension treated with the nanomaterial solvent represented the positive control (CTR+). A volume of 125 μL of the bacterial culture, corresponding to 1 × 10^6^ CFU mL^−1^ (5 × 10^5^ CFU well^−1^), was inoculated in each well. The antimicrobial activities of the compounds were evaluated after 3 h for AgNPs and 3 h under continuous light exposure (UV-A, two lamps of 8 W, 15 cm distance from the plate, irradiance: 34 mW cm^−2^) for TiO_x_-based photocatalyst exposure at 37 °C. The bacterial suspensions, untreated and treated with nanomaterials, were serially diluted in MH broth. The latter were plated and incubated overnight at 37 °C. The resulting colonies were counted, and CFU per milliliter values were obtained. 

### 2.13. Statistical Analysis

Tests were conducted in biological triplicate, and results were indicated as means ± standard deviation (SD).

## 3. Results

### 3.1. Physicochemical, Morphological, and Optical Characterizations

#### 3.1.1. Raman Spectra Evaluation

**Figure 1:**
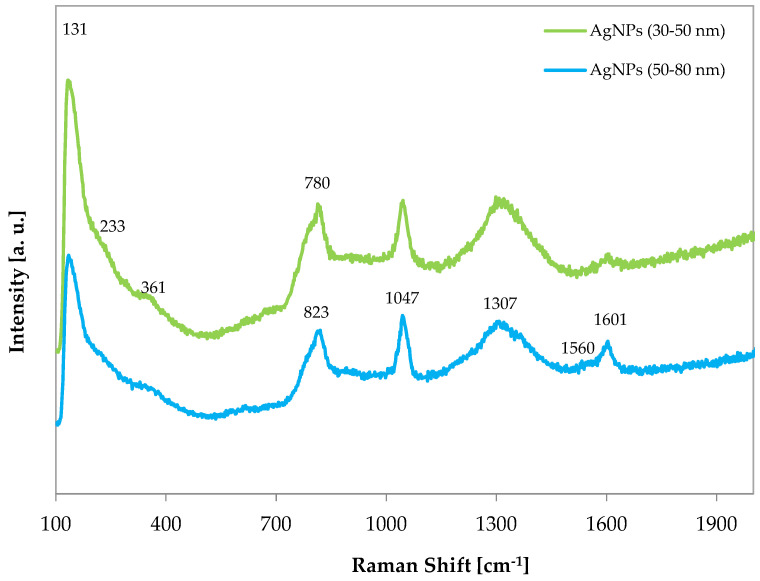
Raman spectra of commercial silver nanoparticles.

In Figure 1, the Raman spectra of AgNP 50–80 and 30–50 nm are presented. The similarity between the two spectra suggests a similar synthesis method. The bands observed in the spectra of the AgNPs appear broadened, and some shoulders are noticeable, indicating the presence of adsorbed species. Because the NPs are exposed to the atmosphere, several bands are detected. The first signal is identified at 131 cm^−1^. A detailed study for the identification of Raman bands of silver compounds and surface species has been conducted in the work of Martina et al., and the following attributions will be based on it [39]. Starting from the T’ modes of the Ag^+^ cation, they are found at 98 (strong) and 146 (shoulder) cm^−1^, and some ionic silver can be present in AgNPs by comparison with the signal at 131 cm^−1^ reported herein. The shoulder at 233 cm^−1^, observed in the spectra of both AgNPs, could be attributed to low amounts of Ag_2_O by v (Ag–O_2_) found at 240 (strong) cm^−1^ in reference sample’s spectrum. The band at 361 cm^−1^ in the AgNP spectrum could also be assigned to the presence of the silver oxide. Moreover, a silver-related band can be found as a shoulder at 780 cm^−1^, as indicated by Agarwal et al. [40]. Ag nanoparticles seem to be obtained by the chemical reduction of the silver nitrate solution in the presence of an organic reductant, as suggested by the Raman band at 1047 cm^−1^, which is the main band in the AgNO_3_ powder’s spectrum. The Raman spectra of silver sulfate (Ag_2_SO_4_) instead shows peaks at 455 and 968 cm^−1^, which are not observed in the Raman bands of the AgNPs, indicating its absence. The Raman band in AgNPs’ spectrum at 823 cm^−1^ could indicate the presence of some Ag_2_CO_3_, which can be identified in the range 804–853 cm^−1^ [41]. Additionally, a very broad band is observed at around 1307 cm^−1^ in both AgNPs’ spectra, accompanied by signals at about 1601 and 1560 cm^−1^, with the latter being more pronounced in the spectra of the AgNPs with sizes of 50–80 nm. These bands could likely be attributed to the presence of residual carbon because the D and G bands can shift from their original positions, from 1348 and 1580 cm^−1^ for graphene oxide (GO) [42] to around 1359 and 1601 cm^−1^ for the nanocomposites.

**Figure 2:**
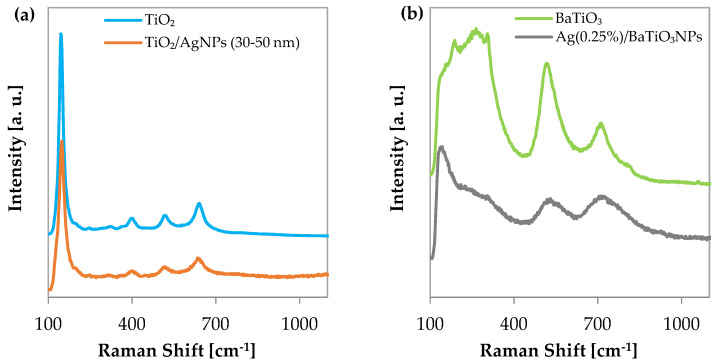
(**a**) Raman spectrum of TiO_2_; (**b**) Raman spectra of BaTiO_3_ and Ag (0.25%)/BaTiO_3_.

In Figure 2a, the Raman spectra of the pristine TiO_2_ and TiO_2_/AgNP (30–50 nm) nanocomposite are depicted. Raman peaks are observed in both spectra at 147 cm^−1^, with a shoulder at around 200 cm^−1^; at 245 cm^−1^, 323 cm^−1^, and 363 cm^−1^ as weak signals; and at 397 cm^−1^, 517 cm^−1^, and 634 cm^−1^. The formed TiO_2_/AgNP 30–50 nm is primarily composed of anatase, characterized by main signals at 144, 404, 526, and 645 cm^−1^ along with a weak shoulder at 201 cm^−1^. The shifts of the Raman signals for the sol–gel TiO_2_ [34] can be attributed to slight differences in the crystalline structure because of the synthesis method, involving sonication.

The low-intensity bands at 245 and 323 cm^−1^ can be attributed to the presence of a small amount of brookite, which spectrum exhibits main signals at 152, 246, and 545 cm^−1^, along with a missing A1g mode at around 320 cm^−1^. However, not all these signals are visible because of overlapping with the strong anatase signals. For a detailed attribution of the Raman bands, refer to [35]. 

The Raman spectra of the pristine BaTiO_3_ NPs and photodeposited Ag/BaTiO_3_ samples are displayed in Figure 2b. The transverse optical modes of the A1 symmetry of the cubic phase are associated with bands at about 517 and 263 cm^−1^ along with a band at 185 cm^−1^, while the B1 mode, linked to the band at around 305 cm^−1^, indicates of the tetragonal BaTiO_3_ phase [43]. The A1(LO) phonons, correlated to the 721 cm^−1^ line, were also connected to the tetragonal lattice. As a consequence, a mixed cubic–tetragonal phase is observed [44,45]. Ag/BaTiO_3_ shows similar bands but with decreased intensity. In particular, a peak at 141 cm^−1^ becomes stronger, attributed to the presence of AgNPs in ionic form, while both bands associated with cubic and tetragonal phases diminish, with a stronger decrease for the cubic phase. This could be attributed to the predominant Ag covering of the BaTiO_3_ NPs’ surface, as no thermal treatment was applied after the silver photoreduction.

#### 3.1.2. UV-Vis DRS Spectra Assessment

**Figure 3:**
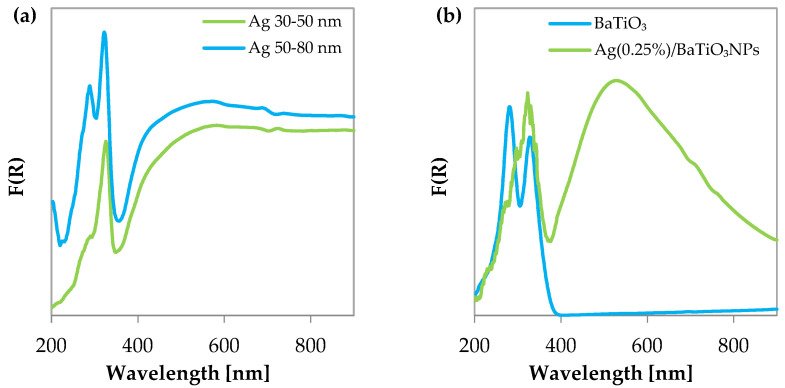
Kubelka–Munk spectra of (**a**) AgNPs and (**b**) BaTiO_3_ NPs and Ag (0.25%)/BaTiO_3_.

UV-vis spectroscopy is one of the most useful methods for characterizing the optical responses of metal nanoparticles, like AgNPs, providing indications about the presence of ionic silver and silver clusters of different charges. It should be noted that the position of SPRs depends on the size, shape, and other factors. In Figure 3a, AgNP 50–80 nm and AgNP 30–50 nm show a broad and very large optical response (surface plasmon resonance, SPR) in the visible region, in agreement with Singaravelan et al. [46]. SPRs were observed up to 600 nm, indicating that most of the obtained AgNPs have different sizes and shapes. For instance, the characteristic sharp SPR formation for colloidal silver nanoparticles was found in the range 350–600 nm [47]. The in-plane dipole plasmon resonance of AgNPs (synthesized in the presence of polyvinyl pyrrolidone, sodium citrate as a capping agent, H_2_O_2_ as an etching agent, and sodium borohydride as a reductant) was observed at 600–800 nm [48]. As can be seen in the UV-vis DRS spectra, the AgNP 50–80 nm presents almost clear bands at 208, 288, and 323 nm, while a shoulder can be difficult to individuate at 480 nm, and the higher absorption is at 575 nm. Furthermore, the presence of a band 323 nm has been identified to correspond to 56 nm AgNPs, in agreement with the size declared by the supplier [48]. According to the literature [49], the band occurring between 200 and 240 nm is related to the presence of Ag^+^ cations in different environments. Interestingly, other signals, although more difficult to assign, can be compared with the absorptions of charged and metallic clusters (Ag_n_^δ+^ and Ag_m_°, where n and m are small indices), which can be assumed to lie between 240–280 nm and 280–350 nm, respectively. As a consequence, the bands at 288 and 323 nm can be attributed to the partial reduction of Ag^+^ cations during the synthesis [50]. Concerning AgNP 30–50 nm, it is relevant to note that the absorptions at around 208 and 288 nm are absent; meanwhile, the most important UV band can be found at 326 nm. This indicates that a more complete reduction of the silver precursor was realized, and small clusters of metallic silver are present. In addition, more evident bands at 353 and 381 nm can be detected as shoulders, attributed again to reduced Ag clusters of zerovalent silver. Generally, the absence of a peak at about 300 nm for silver nitrate testifies that Ag^+^ was almost completely reduced to Ag^0^ [51]. 

In Figure 3b, barium titanate absorptions obtained from UV-vis-DRS spectra are reported. Characteristic bands are found at around 278 nm and 332 nm; all the signals are in the UV field. The onset of the UV absorption is at 391 nm, in agreement with [52], and corresponds to (Planck’s law [53], Eg = hc/λ) a bandgap of 3.17 eV. From the Tauc plot (Figure 5e), it is possible to observe the intercept of the linear part of the transformed signal at 3.15 eV, in very good agreement with the previous estimation.

The absorption bands differ from those of the large-size BaTiO_3_ nanoceramic [54], for which the maximum is found at 300 nm for a particle size of about 900 nm. However, thin films of BaTiO_3_ display bands in the range 300–335 nm [55], indicating that the 100 nm particles are dominated by minute crystallites. In the presence of AgNPs obtained by photodeposition, the BaTiO_3_ band at 278 nm appears decreased, and in addition, an absorption at 304 nm could be observed, attributed to Ag nanoparticles. The band at 332 nm appears stronger and is complicated by very sharp signals, which cannot be attributed for sure to silver nanoparticles. However, it is clearly observed in the visible part of the AgBaTiO_3_ spectrum, a large absorption centered at around 530 nm, as observed in the spectra of Ag nanoparticles 30–50 nm and attributed to their SPR. Figure 3b makes it evident that the AgBaTiO_3_ spectrum displays a unique and single SPR location in the 400–900 nm region, suggesting that the majority of the AgNPs sizes is less widely distributed [47]. 

**Figure 4:**
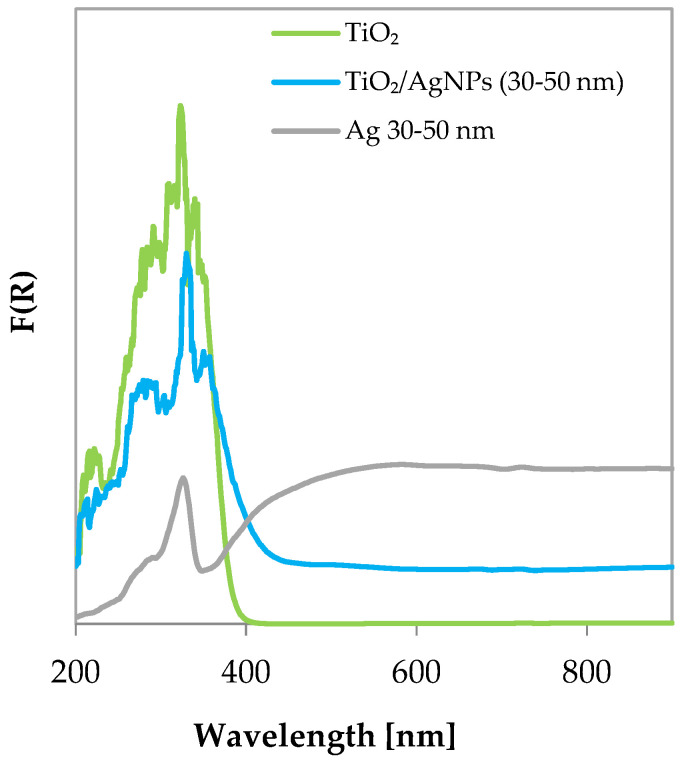
Kubelka–Munk spectrum of TiO_2_/AgNPs compared with those of the single compounds.

**Figure 5:**
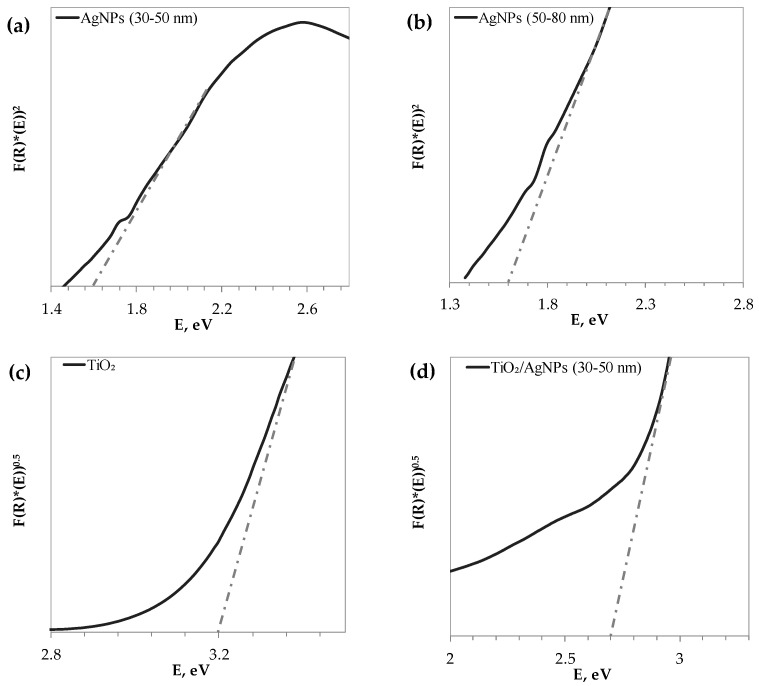

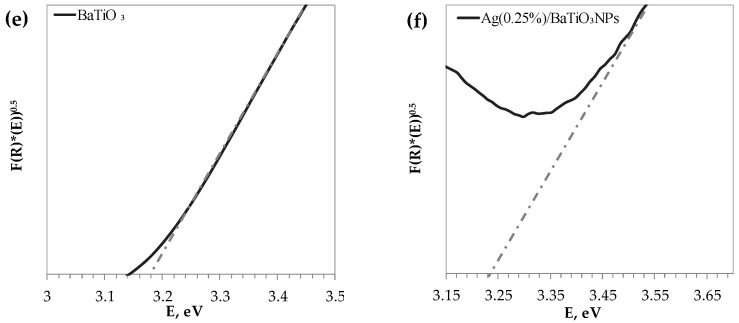
Tauc plots for determining the optical bandgaps of the samples, (**a**) AgNP (30–50 nm), (**b**) AgNP (50–80 nm), (**c**) TiO_2_, (**d**) TiO_2_/AgNP (30–50 nm), (**e**) BaTiO_3_, (**f**) Ag (0.25%)/BaTiO_3_.

Figure 4 reports the K-M absorptions of pristine TiO_2_, TiO_2_/Ag (30–50 nm), and AgNP 30–50 nm. The most prominent band for TiO_2_ is at 321 nm, and all the bands fall within the UV range. For TiO_2_/Ag (30–50 nm), the major band appears at 331 nm, which, upon comparison with the specific absorptions of pristine silver nanoparticles, can be assigned to their presence in the composite. The absorption edge is shifted, indicating the near-UV–visible light activation of the photocatalyst. Figure 5 shows the Tauc plots for the determination of the energy bandgaps of the samples. In particular, the bandgap values are reported in Table 1. The obtained values are in agreement with the values reported in the literature [56,57,58].

#### 3.1.3. TGA-DTG-DSC, SSA, and XRD Characterizations of Nanomaterials

In Figure S2, the TGA, DTG, and DSC signals related to the different nanomaterials are reported. All the samples exhibit small total weight losses, less than 5%, by endothermic-associated signals. A comparison between TiO_2_ obtained by the sonicated sol–gel (Figure S2a) method with a sol–gel TiO_2_ shows (Figure S2b) that both present a first weight loss of about 3% because of the release of physisorbed water, followed by a second complex weight loss in the range 150–600 °C, attributed to the collapse of the different hydroxyl groups present by releasing H_2_O. This second loss is larger (about 2.2%) for sonicated TiO_2_ with respect to TiO_2_ without sonication (1.5%), indicating the presence of different kinds of terminal and bridged hydroxyls, so sonicated TiO_2_ can be considered as a nanomaterial having a higher surface disorder. The TiO_2_/AgNP composite shows a similar behavior, with the second step being less complex, even though sonicated synthesis was performed, corresponding to a 1.4% weight variation. The AgNPs (Figure S2c,e) display two main steps for the release of residual compounds by their synthesis, as individuated in the Raman analysis. The decomposition of the nitrate and carbonates may occur, accompanied by endothermic peaks. Finally, a comparison among BaTiO_3_ and Ag (0.25%)/BaTiO_3_ (Figure S2f) shows that no modification of the weight losses or heat signals is attributed to the photodeposition of silver nanoparticles on BaTiO_3_ surface.

The SSA values of the studied nanomaterials are reported in Table 1. For AgNPs, the surface area is lower than that calculated from their declared dimensions, indicating the presence of aggregates of non-porous nanoparticles. Considering that Ag’s density is 10.5 g/cm^3^, pseudospherical particles have a mean diameter (D) of 65 nm, and SSA = 6/*ρ*D, the SSA values are equal to 14.3 and 8.8 m^2^/g for AgNP 30–50 nm and 50–80 nm, respectively. In the cases of BaTiO_3_ and Ag (0.25)/BaTiO_3_, a similar calculation with a mean dimension of 100 nm and a density of 6.02 g/cm^3^ gives a value of about 10 m^2^/g, which is in agreement with the SSAs of 11 and 12 m^2^/g found for the studied sample, respectively. For TiO_2_, it is possible to calculate a mean size dimension of 14.8 nm using the previous formula. When the composite TiO_2_/Ag is formed, the area had a small decrease, indicating that the smaller nanoparticles of TiO_2_ surround and include the AgNPs. The very different values of SSA among the different TiO_x_-based materials could affect the photocatalytic activity, even if low-surface-area photocatalysts, such as ZnO, show the same relevant photoefficiencies [59]. In general, a higher surface area causes improved efficiency, increasing pollutant adsorption capacity and resulting in more active sites for photocatalytic reactions, at parity with the photoabsorption property, crystalline phase, exposed crystal facets, secondary particle size, etc. [60]. 

**Figure 6:**
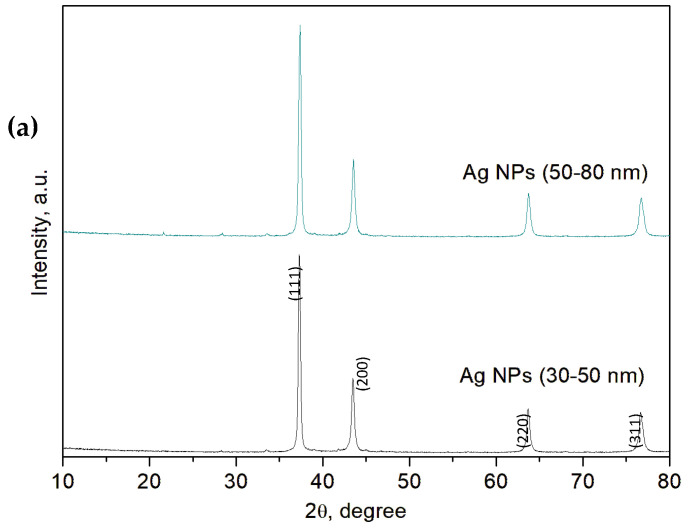

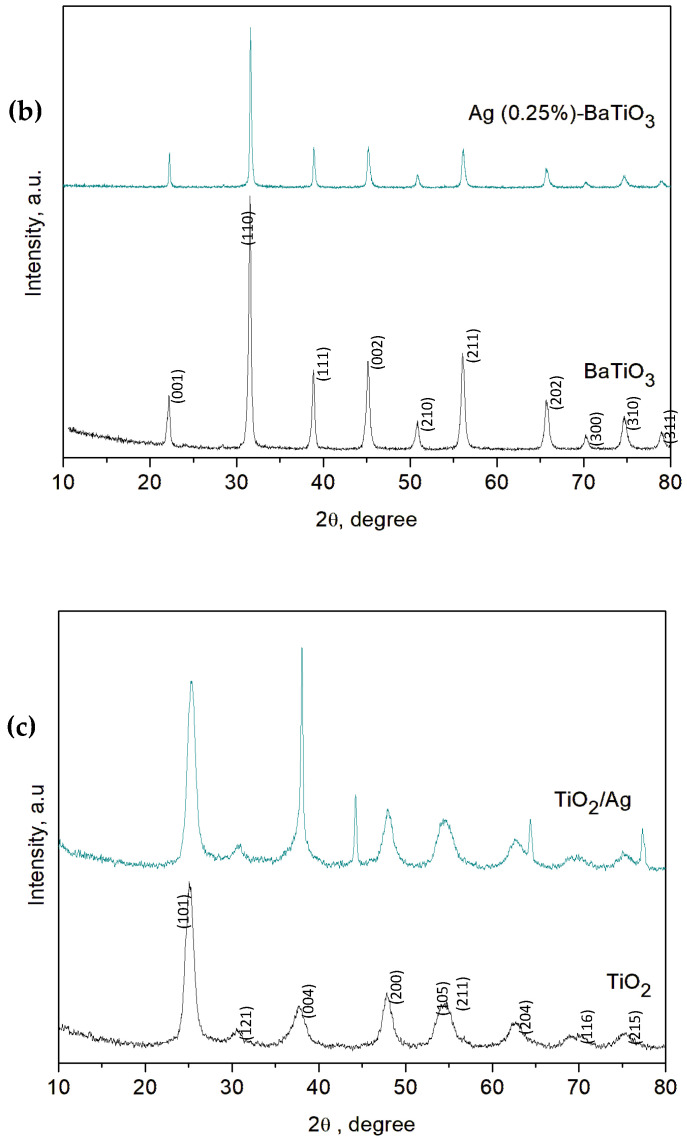
(**a**) X-ray diffraction pattern of Ag nanoparticles; (**b**) X-ray diffraction patterns of Ag (0.25%)-BaTiO_3_ and BaTiO_3_; (**c**) X-ray diffraction patterns of TiO_2_/Ag and TiO_2_.

XRD analysis was utilized to determine the crystallinity, crystallite size, and phases of the powder material. Figure 6 reports the X-ray diffraction patterns (XRD) of Ag, Ag (0.25%)-BaTiO_3_, BaTiO_3_, TiO_2_/Ag, and TiO_2_ nanoparticles. In Figure 6a, the AgNPs’ spectra illustrate the indexed and assigned peaks, with their related positions, such as (111) 38.03°, (200) 44.17°, (220) 64.29°, and (311) 77.19°. These peaks correspond to the face-centered cubic structure of silver (JCPDS file No. 04-0783) [61]. The nanoparticle size was found to be in the range 30–50 nm for the spectrum below and 50–90 nm for the other one [62].

Figure 6b depicts the comparison between the XRD spectra of Ag (0.25%)/BaTiO_3_ and BaTiO_3_. The XRD spectrum of BaTiO_3_ shows peaks at 22.14°, 31.5°, 38.8°, 45°, 50.8°, 56.1°, 66.1°, 70.3°, 74.79°, and 79.01°, which were ascribed to (001), (110), (111), (002), (210), (211), (202), (300), (310), and (311) planes, respectively (JCPDS No. 01-089-1428) [36,63]. The Ag (0.25%)/BaTiO_3_ spectrum is quite similar to the previous one because the low silver load does not allow the distinction of its peak positions with respect to those of barium titanate [62]. 

Figure 6c illustrates all the diffraction peaks in the XRD spectra related to TiO_2_/AgNP (30–50 nm) and TiO_2_ alone. Starting from TiO_2_, the diffraction peaks for the anatase phase could be observed at 2θ = 25.23°, 37.71°, 47.72°, 54.16°, 55.32°, 62.54°, 70.4°, and 75.1°. These peaks were well indexed to the corresponding tetragonal crystal planes (101), (004), (200), (105), (211), (204), (116), and (215), respectively (JCPDS No. 73-1764) [64,65,66]. Furthermore, at 2θ = 30.81°, the characteristic peak relative to TiO_2_ in the brookite phase was recorded, which was ascribed to (121) [67]. Instead, considering the spectrum of TiO_2_/AgNP (30–50 nm), some distinct peaks of silver were detected at 48.17°, 64.29°, and 77.19° [62]. The crystallite sizes of the TiO_2_ obtained by sonicated sol–gel synthesis were similar (around 7 nm) to those reported in [68] for sol–gel synthesis.

### 3.2. Results of Photocatalytic Activity Tests in the MB Mineralization

**Figure 7:**
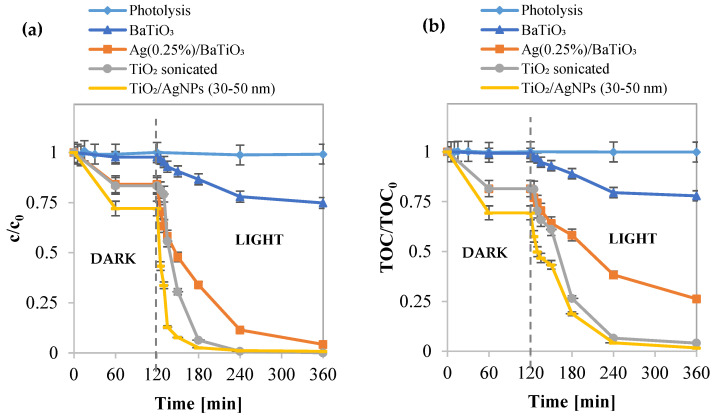
(**a**) Photocatalytic discoloration and (**b**) photocatalytic mineralization of BaTiO_3_, Ag (0.25%)-BaTiO_3_, TiO_2_/Ag, and TiO_2_.

**Figure 8:**
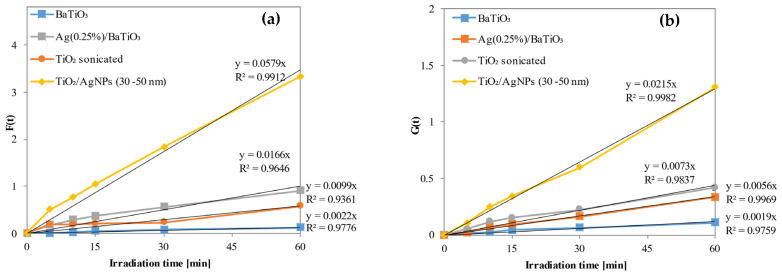
Kinetic plots of the calculated discolorations (**a**) and mineralizations (**b**) for the nanomaterials used in the photocatalytic activity tests.

For the photocatalytic activity tests, concerning the decolorization and mineralization of methylene blue, the following samples were used: BaTiO_3_, Ag (0.25%)/BaTiO_3_ NPs, TiO_2_, and TiO_2_/AgNP (30–50 nm). Additionally, a photolysis test was conducted to evaluate the impacts of light radiation emitted by different light sources on the degradation of the azo dye. The results, reported in Figure 7a,b, underscore that UV-A light alone does not facilitate the oxidation of the organic pollutant. However, the TiO_2_/AgNP (30–50 nm) photocatalyst exhibits the most efficient MB discoloration and mineralization activities. In Figure 8a,b the kinetic pseudo first order fittings are reported. The best sample demonstrates an MB mineralization reaching 99% after three hours of UV-A light exposure, with a kinetic constant of discoloration valued at 0.0166 min^−1^. 

Additionally, the results of the photocatalytic activity tests reveal that the inclusion of silver in the samples accelerates the mineralization of the azo dye, leading to a significant increase in the mineralization kinetic constant (Table 2). 

This enhancement occurs because metallic silver acts as an electron trap and forms a Schottky barrier at TiO_2_–metal junctions. Consequently, the presence of silver facilitates interfacial charge transfer and impedes the recombination of electron–hole pairs, thereby expediting the mineralization process [69,70,71]. 

### 3.3. Evaluation of Degradation Mechanism of MB

Subsequently, photocatalytic activity tests were conducted to investigate the reaction mechanism governing the degradation process of the dye, employing a fixed initial concentration of specific scavenger molecules. The experimental tests were realized using an initial MB concentration equal to 7 ppm, and a TiO₂/AgNP (30–50 nm) dosage of 3 g L^−1^. The scavengers used were the following: isopropanol (IPA, 10 mM) for hydroxyl radicals (${OH}^{•}$) [72], benzoquinone (BQ, 1 µM) for superoxide radicals ($O_{2}^{-•}$) [73], and ethylenediaminetetraacetic acid sodium (EDTA, 10 mM) for positive holes ($h^{+}$) [74]. 

**Figure 9:**
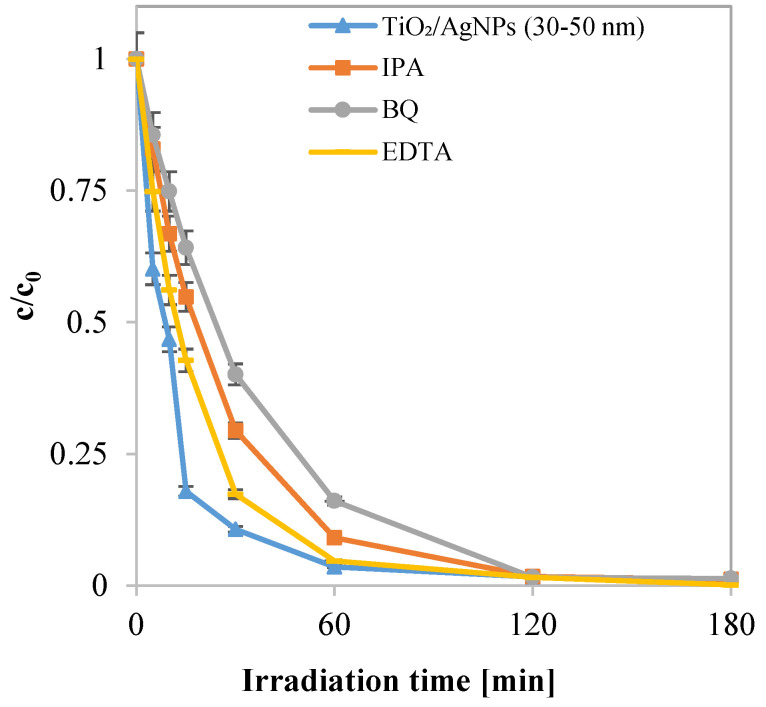
Trend of normalized MB concentration with respect to its initial value as a function of irradiation time, obtained for the tests under the optimal conditions in the presence of IPA, BQ, and EDTA and without scavengers.

**Figure 10:**
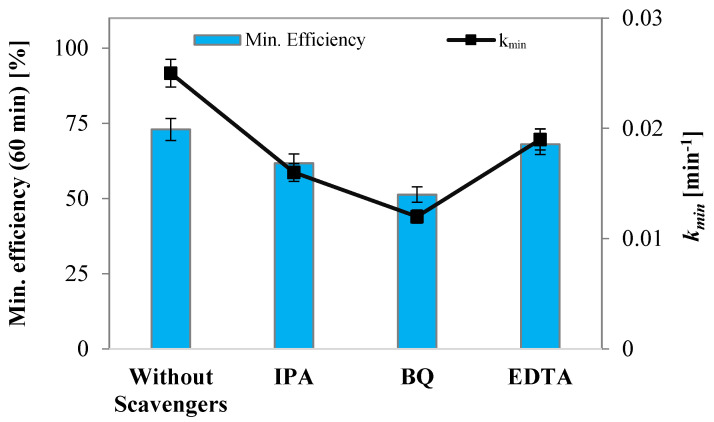
Mineralization efficiency values after 60 min under UV-A light and apparent mineralization kinetic constants obtained for the tests in the presence of IPA, BQ, and EDTA and without scavengers.

The results obtained from these tests are shown in Figure 9 and Figure 10, revealing a significant decline in photocatalytic activity when hydroxyl radicals and superoxide radicals are not formed. Indeed, the tests conducted with IPA and BQ led to reductions in the apparent discoloration kinetic constants of 36% and 52%, respectively. Furthermore, a minor decrease in photocatalytic activity was observed in the test in the presence of EDTA. This outcome can be attributed to the removal of positive holes from the system, resulting in a decrease in hydroxyl radical formation. However, simultaneously, this action hinders the recombination reaction of the photoexcited pairs, which explains the marginal decrease in activity [75].

A possible reaction mechanism that describes the mineralization of the pollutant is constituted by the following reactions:

(10) $$TiO₂/AgNP\;(30–50\;nm)+h\;\nu \to h^{+}+e^{-}$$

(11) $$O_{2}+e^{-}\to O_{2}^{-•}$$

(12) $$OH^{-}+h^{+}\to OH^{•}$$

(13) $$MB+OH^{•}/O_{2}^{-•}\to intermediates\to CO_{2}+H_{2}O$$

(14) $$e^{-}+h^{+}\to E+N$$

where N is a neutral center, and E is the energy generated by the recombination of the photoexcited pairs (light $h\;\nu^{\prime }\leq h\;\nu$ or heat).

### 3.4. Electrical Energy Consumption Comparison

The electrical energy consumption related to the photodegradation of 90% of the MB in 1 m^3^ of water contaminated by the pollutant was calculated for the test with the optimal photocatalyst. For this evaluation the following correlation by Bolton et al. was used [76]:

(15) $$E_{E/O}=\frac{P\;t_{90\%}\;1000}{V\;60\ln\left(\frac{c\left(t_{0}\right)}{c\left(t\right)}\right)}$$

where *P* is the nominal power of the light source (kW), *t*_90%_ is the irradiation time to obtain the 90% removal of the MB (min), *V* is the volume of the treated solution (L), *c*(*t*_0_) is the MB concentration at the initial irradiation time (ppm), and *c*(*t*) is the MB concentration at the irradiation time *t* (ppm). 

Furthermore, the following works have been individuated in the literature on the degradation of methylene blue in aqueous solutions using a photocatalytic process under UV irradiation:

(1) W1 [77]: the photocatalytic reaction was conducted in a photocatalytic reactor under ultraviolet and visible light irradiations using a 100 W mercury lamp (253 nm) and a 100 W LED (589 nm). A photocatalyst weight equal to 200 mg (Cu-TiO_2_/50-C_3_N_4_) was added to 0.1 L of an MB aqueous solution (10 ppm, initial concentration);
(2) W2 [78]: the photocatalytic performances of Bi@SiNWs nanocomposites were evaluated by the degradation of MB under UV light irradiation. All the experiments were performed in a photoreactor irradiated by a UV-C tube lamp (Philips, 55 W). In each test, the photodegradation of 20 mL of the MB solution, with an initial dye concentration of about 3.2 ppm, was analyzed;
(3) W3 [79]: the photocatalytic activity of TiO_2_ (P25) was evaluated in a photocatalytic reactor under visible light (six lamps, Philips Actinic BL, 11 W). Photocatalysts with a loading of 0.1 g L^−1^ were added to 50 mL of the MB solution (initial concentration, 4 ppm), and the irradiation time was equal to 120 min;
(4) W4 [80]: a slurry reactor with a suspension composed of (Nb_0.5_Si_0.5_)_x_Ti_1−x_O_2_ nanocomposites was irradiated by the use of a solar simulator (model ORIEL SOL-2A, 1000 W), and the volume of the MB aqueous solution was 0.002 L;
(5) W5 [81]: degradation runs were carried out in 100 mL of an MB aqueous solution, with an initial concentration of the pollutant equal to 10 ppm, using an α-Fe_2_O_3_ NP dosage equal to 0.1 g L^−1^ and irradiating the system with a UV-A lamp (5 W);

Subsequently, the apparent discoloration kinetic constant of the MB was determined. Additionally, the electricity consumption values needed for a 90% reduction in the pollutant in 1 m^3^ of the solution were calculated using Equation (15) and compared to those required by the reaction system employed in this study under optimal conditions. The results are presented in Table 3.

From the comparison with the literature, it is evident that the photocatalyst made up of silver nanoparticles with titania and irradiated by UV-A light shows a significant reduction in the energy consumption for the treatment of the aqueous solution contaminated by the dye (43 kWh m^−3^).

### 3.5. Antibacterial Activities of Nanomaterials

The antibacterial activities of the AgNPs with sizes of 30–50 nm and 50–80 nm and of TiO_2_/AgNP (30–50 nm) and Ag (0.25%)/BaTiO_3_ under UV irradiation were assessed against standard *E. coli* and *S. aureus* strains. Both AgNP suspensions exhibited robust dose-dependent antibacterial properties. AgNPs with a diameter of 30–50 nm inhibited the growth of *E. coli* by 2.4 × 10^9^, 3.2 × 10^4^, 4.6, and 0.9 times at concentrations of 500, 100, 50, and 10 µg/mL, respectively (Table 4). Similarly, against the same bacterial strain, AgNPs with a diameter of 50–80 nm reduced the bacterial load by 1.1 × 10^10^, 1.9 × 10^8^, 1.6 × 10^3^, and 0.71 times at the same concentrations, compared to the unexposed control. The growth of *S. aureus* (Table 5) decreased by 3.4 × 10^9^, 6.3 × 10^4^, 2.7, and 1.2 times compared to the control, in response to exposure to 30–50 nm AgNPs at doses of 500, 100, 50, and 10 µg/mL, respectively. Otherwise, reductions in the bacterial loads of 1.1 × 10^9^, 7.6 × 10^3^, 2.8, and 1.4 times were achieved when the bacterial inoculum was exposed to 50–80 nm AgNPs at concentrations of 500, 100, 50, and 10 µg/mL, respectively. Lower antibacterial potentials were found for TiO_2_/AgNP (30–50 nm) (UV) and AgNP (0.25%)/BaTiO_3_. The first reduced the growths of *E. coli* and *S. aureus* by 487.5, 186.3, 20.8, and 10.4 times and by 1.1 × 10^3^, 159.2, 90.7, and 45.3 times in response to treatment with 30–50 nm AgNPs at concentrations of 500, 100, 50, and 10 µg/mL, respectively. Regarding Ag (0.25%)/BaTiO_3_, the growth of *E. coli* was impaired by 8.7, 4.7, 1.1, and 0.3 times compared to the untreated control when exposed to the nanomaterial at concentrations of 500, 100, 50, and 10 µg/mL. However, lower antibacterial efficiency was observed using BaTiO_3_ alone. In detail, reductions in bacterial growth of 7.6, 3.9, 1, and 0.2 times compared to the untreated control occurred in response to treatment. For *S. aureus*, Ag (0.25%)/BaTiO_3_ reduced the bacterial load by 228.9, 171.5, 100.2, and 19 times compared to the untreated bacterial suspension at the same doses, respectively. Nonetheless, the antibacterial potential of BaTiO_3_ was inferior to its Ag (0.25%)/BaTiO_3_ counterpart, as evidenced by a reduction in the *S. aureus* burden by factors of 25, 3.3, 2.1, and 1.3 times after exposure to the nanomaterial.

## 4. Discussion

The escalation of antibiotic resistance among pathogenic bacteria has emerged as a pressing global concern, forcing the exploration of effective alternatives to prevent their proliferation. Recent scientific revelations highlight the critical role played by inanimate surfaces exposed to sunlight in facilitating the spread of multidrug-resistant nosocomial pathogens. In this context, the use of AgNPs for surface functionalization has garnered considerable attention, thanks to their distinctive physicochemical attributes and intrinsic antimicrobial ability. Despite the robust antimicrobial properties inherent to AgNPs, their practical application encounters challenges, such as reduced stability, toxicity issues, propensity for aggregation, and the potential to serve as bacterial reservoirs. Addressing these limitations is critical to optimize the utility of AgNPs in various applications. A promising strategy to overcome these well-known obstacles involves the incorporation of AgNPs with composite materials, in particular, TiO_2_ and BaTiO_3_. This fusion not only serves to improve the stability of AgNPs but also mitigates issues related to toxicity and aggregation. Furthermore, it confers continuous catalytic activity under sunlight, which contains UV radiation (5% of the solar emission). Additionally, the composite material serves as a barrier against AgNPs functioning as bacterial depots, presenting a comprehensive solution to the challenges associated with standalone AgNP applications. In our investigation, we evaluated the antibacterial efficacy of two distinct AgNP suspensions, one characterized by a size range between 30 and 50 nm and the other between 50 and 80 nm. When faced with *S. aureus*, AgNPs exhibited significant antibacterial activity, resulting in reductions in bacterial loads of 3.4 × 10^9^ and 1.1 × 10^9^ times compared to the untreated control for 30–50 nm and 50–80 nm AgNPs, respectively. Even in the case of *E. coli*, both vesicular suspensions gave similar antibacterial efficacies. In detail, 50–80 nm AgNPs inhibited bacterial replication by 1.1 × 10^10^ times, while the vesicle suspension with a smaller diameter reduced the bacterial population by 2.4 × 10^9^ times compared to the untreated control. Differences in the antibacterial potentials between the two suspensions of AgNPs of different diameters may not be evident after a 24 h period of exposure to Gram-positive and Gram-negative bacterial strains. It is plausible that that disparities may have become apparent with shorter exposure durations. Skandalis et al. employed *Arbutus unedo* leaf extract for the fabrication of silver nanoparticles (AgNPs) with sizes of 58 nm and 40 nm [82]. Their investigation into the antibacterial properties against *E. coli* revealed no significant distinction in antibacterial activity between the 58 nm and 40 nm variants following a 24 h exposure period. However, with longer monitoring durations, it became evident that the 40 nm AgNPs caused damage to the bacterial membrane after 10 h, whereas the 58 nm AgNPs achieved a similar effect after 24 h [83]. There are various pieces of evidence proving the antibacterial potential of AgNPs. Regardless of the synthesis method that was employed, AgNPs demonstrate antibacterial efficacy, albeit with varying degrees of potency. Loo et al. employed pu-erh tea leaf extract as a silver-reducing agent in the synthesis of AgNPs. The antibacterial efficacy of these synthesized AgNPs was evaluated against a broad spectrum of food-borne Gram-negative pathogens. The obtained data revealed minimal inhibitory concentration (MIC) and minimal bactericidal concentration (MBC) values of AgNPs against *E. coli*, *Klebsiella pneumoniae*, *Salmonella typhimurium*, and *Salmonella enteritidis*, respectively, to be 7.8, 3.9, 3.9, and 3.9 μg/mL and 7.8, 3.9, 7.8, and 3.9 μg/mL, demonstrating bactericidal activity [84]. Jabbar et al. investigated the antibacterial efficacy of silver nanoparticles functionalized with *Equisetum diffusum* (horsetail) extract against both Gram-positive and Gram-negative bacterial strains. Specifically, the nanoparticles elicited inhibition zones measuring 18 mm and 10 mm for *Listeria monocytogenes* and *Escherichia coli* strains, respectively [85]. Moreover, Quintero-Quiroz et al. assessed the antibacterial activity of AgNPs synthesized via a chemical method, employing NaBH_4_ as a reducing agent for silver [86]. The resulting nanoparticle suspension demonstrated minimum bactericidal concentrations (MBCs) of 19.89, 9.94, and 9.94 μg/mL against *S. aureus*, *E. coli*, and AmpC-resistant *E. coli*, respectively. AgNPs demonstrated relevant antibacterial activities under the conditions set for both bacterial strains, although this may also be because of residual ionic compounds from their production, as demonstrated by Raman spectra and TGA. As previously discussed, to address the limitations associated with the use of AgNPs, we combined them with TiO_2_ and BaTiO_3_. As expected, the latter exhibited a lower antibacterial potential compared to their AgNP counterpart. In fact, less than 25-fold reductions in bacterial loads occurred for both strains. Therefore, to enhance their antibacterial activities and physicochemical characteristics, TiO_2_ and BaTiO_3_ nanomaterials were conjugated with small-sized nanoparticles (30–50 nm), as they possess greater antibacterial potential. It must be remarked that TiO_2_ alone, even with a high SSA, is slightly active, indicating that the presence of silver is essential to obtain higher performances. The superior photooxidation capability of TiO_2_/AgNP (30–50 nm) is also evidenced in the photodiscoloration and mineralization of MB, being almost total after 120 min and 240 min, respectively. In a study by Sagadevan et al., it was found that TiO_2_ at a concentration of 100 µg/mL showed a significant (72%) reduction in *E. coli* levels. Furthermore, the combination of 2.5% AgNP-TiO_2_ produced a strong reduction in the bacterial load of 4.98 log [87]. Several studies have suggested that AgNP-TiO_2_ exhibits antimicrobial properties via two distinct mechanisms: the release of silver ions and direct contact-mediated killing. Zawadzka et al. conducted an assessment of the antimicrobial efficacy of AgNP-TiO_2_ against strains of *Staphylococcus aureus*. Utilizing confocal microscopy, they quantified antibacterial effectiveness and elucidated mechanisms of action by employing both confocal and scanning electron microscopy (SEM), complemented by silver release analysis using atomic absorption spectrometry (AAS). Confocal microscopy analysis revealed that the mortality rates of *Staphylococcus aureus* were lower than those in the inhibition of microbial growth, suggesting a multifaceted antimicrobial activity of the tested nanomaterials. SEM examinations indicated the bactericidal effect of AgNP-TiO_2_ through the formation of pores, leading to the release of cytosolic contents and subsequent bacterial cell lysis. Following exposure, a subset of bacteria demonstrated impaired replication, likely attributable to DNA damage, the inhibition of bacterial cellular respiration, mitochondrial dysfunction, or protein peroxidation induced by silver ions. Indeed, AAS analysis demonstrated a significant increase in silver release, thereby enhancing the efficiency of AgNP coatings against *Staphylococcus aureus* [88]. Cao et al. investigated the biological efficacy of AgNPs embedded within titanium, regulated by a microgalvanic effect, and demonstrated the considerable antibacterial capabilities of the examined composites. Consequently, the authors observed microbial demise attributed to the disruption of the bacterial cell membrane upon contact with AgNPs [89]. In another study, the structural integrity of the outer membrane of *Escherichia coli* was investigated following exposure to AgNP/TiO_2_. Subsequent bacterial cell damage was observed following treatment, as visualized in field emission scanning electron microscopy (FE-SEM) images [90]. Discrete photoactivity is observed for Ag (0.25%)/BaTiO_3_ NPs (UV), which aligns with its lower antibacterial efficacy. In detail, at the maximum dose, it resulted in reductions in the loads of *E. coli* and *S. aureus* by 8.7 and 228.9 times, respectively, under UV conditions. However, the trend observed in the removal of MB through photocatalysis does not correspond to the behavior observed in antibacterial properties, as TiO_2_ exhibits high photoactivity. The emerging bifunctional nanomaterial among those tested is TiO_2_/AgNP (30–50 nm), which achieves a reduction in *S. aureus* of about three orders of magnitude and about one-half with respect to *E. coli* (at a dosage of 500 µg/mL). Meanwhile, it possesses the highest photoactivity in MB degradation. It is believed that with silver embedded inside TiO_2_ nanoparticles, silver acts as an electron withdrawer, aiding in the separation of photogenerated charges and, thereby, improving the availability of oxidizing holes, forming a Schottky junction. The co-catalyst’s ability to act as a reservoir for photoinduced electrons that are transferred from semiconductor surfaces is one of its key functions. This increases the generated ROSs able to degrade bacteria. The obtained results are in line with recent evidence. Yerli-Soylu et al. fabricated fibrous TiO_2_ nanocomposites incorporating AgNPs through a combination of sol–gel and electrospinning techniques. The photocatalytic efficacy of these nanofiber membranes was assessed by monitoring the changes in MB concentrations in aqueous solutions under UV irradiation. Remarkably, the TiO_2_ nanocomposite membrane doped with 2.5% AgNPs exhibited a very high MB degradation rate of 94.6%, highlighting the synergistic effect of TiO_2_ and AgNPs [91]. Likewise, Kodom et al. investigated the impact of the AgNP-TiO_2_ configuration on the visible-light-driven photocatalytic activity. The resulting hybrid material exhibited enhanced photocatalytic performance compared to those of its individual components. Specifically, the hybrid material facilitated the degradation of 45% of the methyl orange after 150 min of exposure to visible light [92]. 

## 5. Conclusions

The combination of composite nanomaterials presents an opportunity for valorizing commercially available AgNPs, thereby mitigating the expenses associated with their use. Characterization results reveal that commercial AgNPs exhibit partial aggregation, along with the presence of unconverted Ag^+^ ions, contributing to their pronounced antimicrobial efficacy and concomitant toxicity. Simultaneously using AgNPs with TiO_2_ or BaTiO_3_ facilitates the development of active photocatalysts, improving their oxidative capabilities. Despite the notable variation in the specific surface areas of TiO_x_ nanomaterials, both demonstrate efficacy in eradicating bacteria under UV light irradiation. However, the sonicated synthesis-derived photocatalyst, TiO_2_/AgNP (30–50 nm), was expected to exhibit dual efficacy in UV photocatalysis for the degradation of methylene blue (MB) and UV-mediated growth inhibition of *E. coli* and *S. aureus*. These cumulative results highlight the potential of the synthesized nanomaterials as promising candidates for applications requiring enhanced antibacterial and photocatalytic performances. The innovation lies in the application of AgNP-TiO_2_-based nano-photocatalysts as disinfectants in healthcare environments. Its unique properties allow for significant reductions in bacterial counts, achieving, in particular, notable decreases of 487.5 and 1.1 × 10^3^ times for *Escherichia coli* and *Staphylococcus aureus*, respectively, at a dose of 500 µg/mL under UV irradiation. This innovative application holds promise for improving hygiene and infection control measures in healthcare environments, potentially reducing the risk of microbial contamination and helping to improve patients’ health.

## Figures and Tables

**Table 1:** Results of specific surface area (SSA), energy bandgap (E_bg_), and average crystallite sizes of nanomaterials (by XRD).

Nanomaterial	SSA, m^2^/g	E_bg_, eV	Crystallite Size, nm
AgNP (30–50 nm)	2.1	1.6	31
AgNP (50–80 nm)	2.2	1.6	26
TiO_2_	95.9	3.14	7
TiO_2_/AgNP (30–50 nm)	89.3	2.7	8
BaTiO_3_	11.9	3.15	28
Ag (0.25%)/BaTiO_3_	12.1	3.32	34

**Table 2:** Apparent values of discoloration and mineralization kinetic constants for the nanomaterials used in the photocatalytic activity tests.

Nanomaterial	*k_disc_*, min^−1^	*k_min_*, min^−1^
BaTiO_3_	0.0022	0.0022
Ag (0.25%)/BaTiO_3_	0.0166	0.0063
TiO_2_	0.0147	0.0055
TiO_2_/AgNP (30–50 nm)	0.0624	0.0248

**Table 3:** Comparison of the energy costs for the reduction of 90% of the MB in 1 m^3^ of solution for our system with TiO_2_/AgNP (30–50 nm) and other works reported in the literature.

Photocatalytic System	Photocatalyst	Type of Light	*V* (L)	*P* (kW)	*k* (min^−1^)	*E_E/O_* (kWh m^−3^)
Our system	TiO_2_/AgNP (30–50 nm)	UV-A	0.1	0.016	0.0624	43
W1	Cu-TiO_2_/50-C_3_N_4_	UV/Vis	0.1	0.2	0.0044	7576
W2	Bi@SiNWs	UV-C	0.02	0.055	0.0183	2505
W3	TiO_2_ (P25)	UV-A	0.05	0.066	0.023	957
W4	(Nb_0.5_Si_0.5_)xTi_1−x_O_2_	UV/Vis	0.002	1	0.131	63,613
W5	α-Fe_2_O_3_ NPs	UV-A	0.1	0.005	0.0128	65

**Table 4:** Reduction in *E. coli* load compared to CTR+. Data are represented as mean fold reductions ± SD.

*E. coli*				
Nanomaterial	500 µg/mL	100 µg/mL	50 µg/mL	10 µg/mL
AgNP (30–50 nm)	2.4 × 10^9^ ± 43,060,989	3.2 × 10^4^ ± 772	4.6 ± 0.2	0.9 ± 0.1
AgNP (50–80 nm)	1.1 × 10^10^ ± 1,679,378,605	1.9 × 10^8^ ± 5,597,928	1.6 × 10^3^ ± 18	0.71 ± 0.2
TiO_2_ (UV)	16.3 ± 5	7.2 ± 1.9	1.7 ± 0.4	0.4 ± 0.01
TiO_2_/AgNP (30–50 nm) (UV)	487.5 ± 17.7	186.3 ± 5.2	20.8 ± 2.6	10.4 ± 0.2
Ag (0.25%)/BaTiO_3_	8.7 ± 1.8	4.7 ± 0.7	1.1 ± 0.08	0.3 ± 0.04
BaTiO_3_	7.6 ± 0.81	3.9 ± 0.23	1.0 ± 0.06	0.2 ± 0.05

**Table 5:** Reduction in *S. aureus* load compared to CTR+. Data are represented as mean fold reductions ± SD.

*S. aureus*				
Nanomaterial	500 µg/mL	100 µg/mL	50 µg/mL	10 µg/mL
AgNP (30–50 nm)	3.4 × 10^9^ ± 53,033,008	6.3 × 10^4^ ± 1645	2.7 ± 0.4	1.2 ± 0.3
AgNP (50–80 nm)	1.1 × 10^9^ ± 530,330,086	7.6 × 10^3^ ± 135	2.8 ± 0.3	1.4 ± 0.2
TiO_2_ (UV)	25 ± 7.1	3.3 ± 0.3	2.1 ± 0.3	1.3 ± 0.4
TiO_2_/AgNP (30–50 nm) (UV)	1.1 × 10^3^ ± 167	159.2 ± 27	90.7 ± 6.1	45.3 ± 3
Ag (0.25%)/BaTiO_3_	228.9 ± 3	171.5 ± 10	100.2 ± 7.4	19 ± 0.3
BaTiO_3_	25 ± 1.4	3.3 ± 0.3	2.1 ± 0.4	1.3 ± 0.2

## Data Availability

Data are contained within the article and Supplementary Materials.

## Supplementary Materials

The following supporting information can be downloaded at: https://www.mdpi.com/article/10.3390/ma17102178/s1, Figure S1: Picture of the experimental apparatus scheme used for the MB mineralization tests; Figure S2: Thermal analyses of nanomaterials.
