# Supplementary material for: Characterization and Photocatalytic and Antibacterial Properties of Ag- and TiOx-Based (x = 2, 3) Composite Nanomaterials under UV Irradiation
Source: Materials (Basel). 2024 May 7;17(10):2178. doi: 10.3390/ma17102178 (PMC11122886; doi:10.3390/ma17102178)
Supplement: Supplementary file 1 [file materials-17-02178-s001.zip › materials-2947918-supplementary.pdf]

SUPPLEMENTARY INFORMATIONS

Characterization and Photocatalytic and Antibacterial Properties of Ag- and TiO_x-Based (x = 2, 3) Composite Nanomaterials under UV Irradiation

Nicola Morante [1,†], Veronica Folliero [2,†], Federica Dell'Annunziata [2,3], Nicoletta Capuano [2], Antonietta Mancuso [1], Katia Monzillo [1], Massimiliano Galdiero [3], Diana Sannino [1,*] and Gianluigi Franci [2,*]

[1] Department of Industrial Engineering, University of Salerno, Via Giovanni Paolo II, 132, 84084 Fisciano, SA, Italy; nmorante@unisa.it (N.M.); anmancuso@unisa.it (A.M.); kmonzillo@unisa.it (K.M.)
[2] Department of Medicine, Surgery and Dentistry, University of Salerno, 84084 Baronissi, SA, Italy; vfolliero@unisa.it (V.F.); federica.dellannunziata@unicampania.it (F.D.); niccapuano@unisa.it (N.C.)
[3] Department of Experimental Medicine, Section of Microbiology and Clinical Microbiology, University of Campania "Luigi Vanvitelli", 80138 Naples, NA, Italy; massimiliano.galdiero@unicampania.it
* Correspondence: dsannino@unisa.it (D.S.); gfranci@unisa.it (G.F.); Tel.: +39-3805817416 (D.S.); +39-3385683762 (G.F.)
† These authors contributed equally to this work.

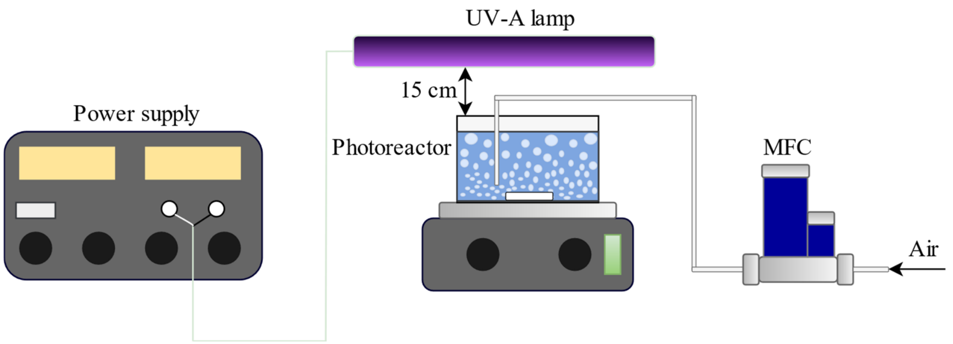

Figure S1. Picture of the experimental apparatus scheme used for the MB mineralization tests.

a)unsonicated TiO_2

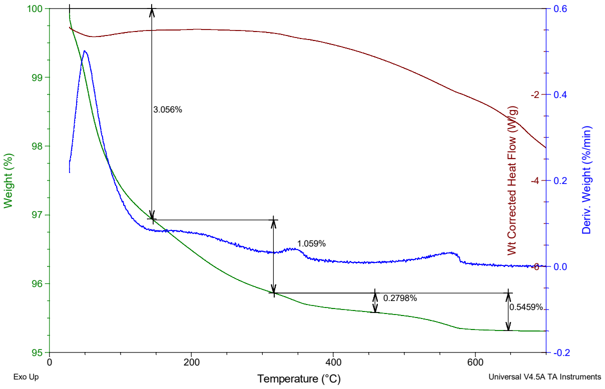

b) sonicated TiO_2

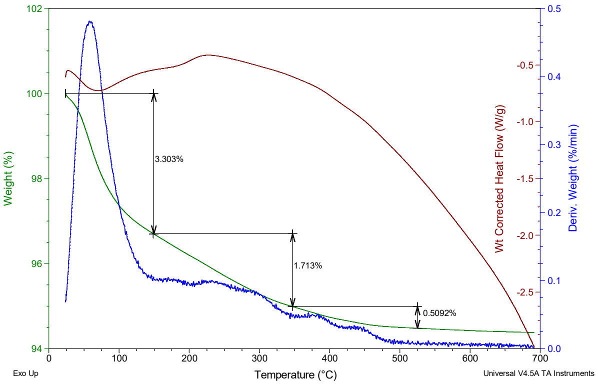

c) Ag NPs50-80 nm

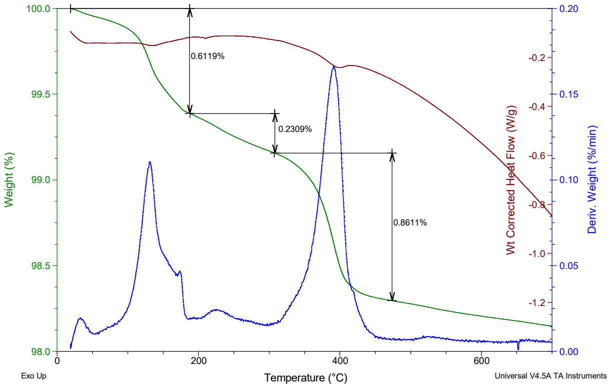

d) TiO_2/AgNPs

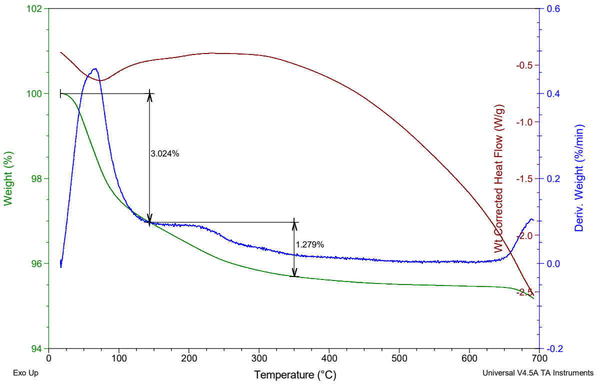

e) AgNPs 30-50 nm

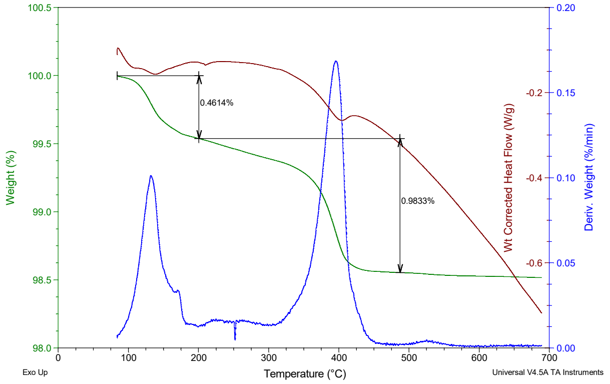

f) Ag(0.25%)$BaTiO_3$ and $BaTiO_3$ NPs

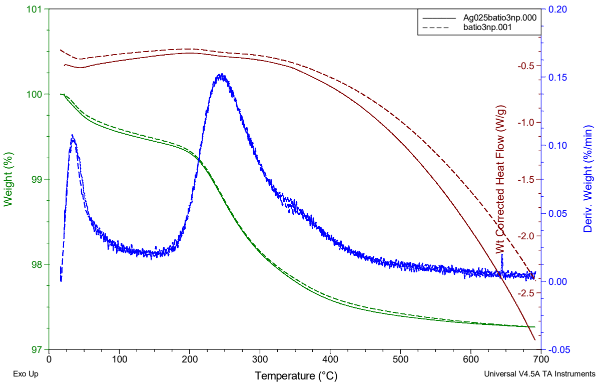

Figure S2. Thermal analyses of nanomaterials.
